# Facile Fabrication of CeO_2_/Electrochemically Reduced Graphene Oxide Nanocomposites for Vanillin Detection in Commercial Food Products

**DOI:** 10.3390/nano10071356

**Published:** 2020-07-11

**Authors:** Xue Nie, Rui Zhang, Zheng Tang, Haiyan Wang, Peihong Deng, Yougen Tang

**Affiliations:** 1 College of Chemistry and Chemical Engineering, Central South University, Changsha 410083, China; hynuniexue@126.com (X.N.); csuzr606@csu.edu.cn (R.Z.); tangzhenggz@csu.edu.cn (Z.T.); wanghy419@126.com (H.W.); 2 Key Laboratory of Functional Metal-Organic Compounds of Hunan Province, Key Laboratory of Functional Organometallic Materials of Hunan Provincial Universities, College of Chemistry and Material Science, Hengyang Normal University, Hengyang 421008, China; dph1975@163.com

**Keywords:** CeO_2_ nanoparticles, vanillin, reduced graphene oxide, nanocomposites, voltammetric determination

## Abstract

In this paper, CeO_2_ nanoparticles were synthesized by the solvothermal method and dispersed uniformly in graphene oxide (GO) aqueous solution by ultrasonication. The homogeneous CeO_2_-GO dispersion was coated on the surface of a glassy carbon electrode (GCE), and the CeO_2_/electrochemically reduced graphene oxide modified electrode (CeO_2_/ERGO/GCE) was obtained by potentiostatic reduction. The results of X-ray diffraction (XRD), energy dispersive X-ray spectroscopy (EDS), scanning electron microscopy (SEM), and transmission electron microscopy (TEM) showed that CeO_2_ nanocrystals were uniformly coated by gossamer like ERGO nanosheets. The electrochemical behavior of vanillin on the CeO_2_/ERGO/GCE was studied by cyclic voltammetry (CV). It was found that the CeO_2_/ERGO/GCE has high electrocatalytic activity and good electrochemical performance for vanillin oxidation. Using the second derivative linear sweep voltammetry (SDLSV), the CeO_2_/ERGO/GCE provides a wide range of 0.04–20 µM and 20 µM–100 µM for vanillin detection, and the detection limit is estimated to be 0.01 µM after 120 s accumulation. This method has been successfully applied to the vanillin detection in some commercial foods.

## 1. Introduction

The problem of food safety has caused public concern all over the world. Unsafe food can lead to many acute and lifelong diseases, from diarrhea to various cancers [1]. Vanillin (4-hydroxy-3-methoxybenzaldehyde), a food additive, is widely used to contribute to the fragrance of various foods, such as ice-cream, cookies, pudding, beverages, custard, and chocolate [2]. However, the production cost of natural vanillin from vanilla pods is very high. Low-cost materials, such as eugenol, 2-methoxyphenol, and lignin, can also be used to synthesize vanillin. Although the synthetic version is cheaper and widely produced, it can lead to headaches, nausea, vomiting, and may affect the functions of the kidneys and liver when large quantities of vanillin are ingested. Therefore, for the sake of human health, the content of vanillin in food should be strictly controlled.

In order to determine vanillin sensitively and effectively, gas chromatography [3], high performance liquid chromatography [4], thin layer chromatography [5], ultraviolet visible spectrophotometry [6], chemiluminometry [7], and capillary electrophoresis [8] were introduced. However, most of these methods need large and expensive instruments, and the operation is complex and time-consuming. The electrochemical method has been one of the research hotspots due to its advantages of easy operation, excellent sensitivity, and low cost. Although vanillin molecules are electrooxidable, the results of direct determination of vanillin with bare electrode are not satisfactory. The main reason is that the fouling and regeneration of electrode surface [2]. In recent years, new electrochemical sensing methods using chemically modified electrodes (CMEs) for vanillin detection have been investigated [9,10,11,12,13,14,15,16,17,18]. For example, Shang et al. developed a vanillin electrochemical sensor based on AuPd-graphene hybrid. They found that the sensor exhibited high electrocatalytic activity towards vanillin oxidation and the electrochemical response was greatly increased [9]. Sivakumar et al. prepared a glassy carbon electrode (GCE) modified with a CoS nanorods–Nafion composite which can be applied for vanillin detection in food samples [10]. Mei et al. reported an electrospun molybdenum disulfide-carbon nanofiber (MoS_2_-CNF) for vanillin detection. In the concentration range of 0.3–135 μM, the peak current shows a good linear response, and the detection limit is 0.15 μM [11]. However, the performance of some modified electrodes is still not ideal due to the poor electrocatalytic activity of the modified materials. Therefore, it is very important to prepare novel modified electrodes for the rapid and effective detection of vanillin.

Graphene (GR) is a kind of planar monolayer structure composed of sp^2^-bonded carbon atoms. With its high specific surface area, unique mechanical and electronic properties, GR has attracted tremendous attention [19]. The properties of GR can be adjusted by surface modification. The organic functionalization and inorganic nanoparticles attachments on the surface of GR significantly changed the physical and chemical properties of GR and greatly expanded its application field [20,21,22,23,24]. Recently, lots of researches have been carried out to modify the surface of GR with MnO_2_ [25,26], SnO_2_ [27], Fe_3_O_4_ [28,29], Co_3_O_4_ [30], Cu_2_O [31,32,33], NiO [34], TiO_2_ [35], and other metal oxide nanoparticles, and to find their applications in electrode modification. CeO_2_ nanoparticles have the advantages of low cost, excellent electrochemical redox characteristic, and environmental friendliness, and are explored as an excellent electrode modifier material [36]. For example, Adarakatti et al. fabricated a GCE modified with mesoporous CeO_2_ nanoparticles and used for the determination of Cu (II) and Hg (II) [37]. Cai and coworkers fabricated an electrochemical immunosensor based on CeO_2_/chitosan nanocomposite for the determination of sulfamethoxazole in foods [38]. Dong et al. synthesized a novel multi-walled carbon nanotubes (MWCNT)–CeO_2_–Au nanocomposite and realized the successful detection of methyl parathion [39]. However, CeO_2_ nanoparticles as an electrode material have some problems, such as easy agglomeration and poor conductivity, which seriously affect its performance in practical applications. Anchoring CeO_2_ on the GR nanosheets with large specific surface area and high conductivity can effectively solve these problems. Some researchers have made great efforts in the preparation of CeO_2_/GR nanocomposites for supercapacitors [40,41]. However, as far as we know, using CeO_2_/GR nanocomposites for electrochemical sensing has not been reported in any literature. In previous reports, most of the methods for CeO_2_/GR preparation are multi-step, involving dangerous or toxic reducing agents, which are prone to environmental and health risks. Furthermore, due to the poor dispersibility of GR nanosheets in the solvents, GR nanosheets tent to agglomerate together by van der Waals interaction and strong π–π stacking, which limit the application in the electrode modification.

In this paper, CeO_2_/graphene oxide (GO) nanocomposites were prepared by the ultrasonic mixing of GO solution and CeO_2_ nanoparticles, and the CeO_2_/ERGO/GCE was obtained by coating the suspension by drop-coating method and further treated by potentiostatic reduction at −1.2 V for 120 s. This method offers several advantages over other techniques for the preparation of CeO2/GR nanocomposite, including being green, efficient, inexpensive, and rapid. The electrochemical behavior of vanillin was investigated on the CeO_2_/ERGO/GCE for the first time. Due to the advantages of each component material and their synergistic effect, the CeO_2_-ERGO nanocomposites significantly improved the performance of the sensor. In addition, the CeO_2_/ERGO/GCE exhibited good reproducibility, high accuracy, and strong anti-interference ability for vanillin detection. More importantly, because the electrode was modified in one step, the electrode fabrication is very convenient and fast. The developed sensor has a wide application prospect in the determination of vanillin in food.

## 2. Experimental

### 2.1. Chemicals

Cerium nitrate hexahydrate was purchased from Chengdu Aikeda Chemical Reagent Co., Ltd. (Chengdu, China). Vanillin, graphite powder, potassium permanganate, sodium nitrate, ethylene glycol, hydrogen peroxide solution (30 wt %), ammonia solution (25 wt %), and hydrazine solution (80 wt %) were obtained from Sinopharm Group Chemical Reagent Co., Ltd. (Shanghai, China). The accurately weighed vanillin was dissolved in a proper amount of ethanol, diluted with water to obtain a 1.0 mM stock solution. Other chemicals used in the experiment are of analytical reagent grade. The water used is ultrapure water.

### 2.2. Apparatus

Electrochemical experiments (cyclic voltammetry and second-order derivative linear sweep voltammetry) were carried out on a chi660e electrochemical workstation (Shanghai Chenhua Instrument Co., Ltd., Shanghai, China) and JP-303E polarographic analyzer (Chengdu Instrument Co., Ltd., Chengdu, China) respectively. A three electrode system was used, i.e., modified GCE with diameter of 3 mm was used as working electrode, and asaturated calomel electrode (SCE) and platinum wire electrode were used as reference electrode and auxiliary electrode respectively. The scanning electron microscope (SEM) images were obtained using a scanning electron microscope (EVO10, ZEISS, Jena, Germany). Transmission electron microscope (TEM) images and energy dispersive X-ray spectroscopy (EDS) were obtained by a transmission electron microscope (JEOL JEM-2100, Tokyo, Japan). The X-ray diffraction data (XRD) were collected on a powder X-ray diffractometer (Rigaku, Tokyo, Japan) (Cu Kα radiation λ = 0.154056 nm). All measurements were performed at room temperature.

### 2.3. Synthesis of CeO_2_ Nanoparticles

CeO_2_ nanoparticles were synthesized via a hydrothermal method. In brief, 1 mL of 0.5 M cerium nitrate aqueous solution was added to 30 mL of ethylene glycol, followed by the addition of 1 mL of ultrapure water, the mixed solution was stirred for 30 min at room temperature and transferred to a 50 mL autoclave for solvothermal reaction at 140 °C for 18 h. After the reaction, the mixture was cooled down to room temperature. The product was centrifuged, washed with water and ethanol, and dried in an oven at 50 °C.

### 2.4. Preparation of GO and CeO_2_-GO Composite

Graphene oxide (GO) was prepared using a modified Hummers method, which involves the steps of graphite oxidation and subsequent exfoliation [42]. A homogeneous CeO_2_-GO dispersion was obtained by mixing 1.0 mg of the CeO_2_ nanoparticles and 1.0 mL GO solution (1 mg mL^−1^) directly by ultrasonication for 20 min.

### 2.5. Fabrication of Modified Electrodes

**Scheme 1:**
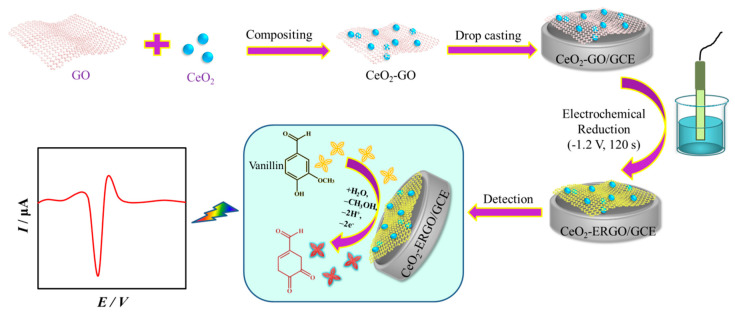
Synthesis of the sensing material and the construction processes of the proposed CeO_2_/ERGO/GCE for vanillin sensing.

Before modification, a GCE was polished on a polishing cloth with 0.3 μm alumina powder, and then ultrasonic treatment was carried out in ethanol and ultrapure water successively. A treated GCE was modified with 5.0 μL of CeO_2_-GO dispersion and the solvent on the electrode surface was evaporated under an infrared lamp. Then the electrode was put into a 0.1 M phosphate buffer (pH 6.5) and reduced at the constant potential of −1.2 V for 120 s to obtain the CeO_2_/ERGO/GCE. For comparison, GO/GCE, CeO_2_/GCE and ERGO/GCE were also fabricated in a similar way. The brief fabrication process of the CeO_2_/ERGO/GCE and its use in vanillin sensing is shown in Scheme 1.

### 2.6. Electrochemical Measurement

For electrochemical measurements, 10 mL 0.10 M HCl solution containing a certain concentration of vanillin was added to a 10 mL cell, and a three-electrode system was installed in the test solution. After accumulation at 0.1 V for 120 s, the cyclic voltammograms was recorded from 0.1 V to 1.2 V and the second-order derivative linear sweep voltammograms were recorded from 0.6 V to 1.2 V. After every measurement, the CeO_2_/ERGO/GCE was regenerated by two successive voltammetric sweeps in 0.6–1.2 V in 0.1 M H_2_SO_4_ solution. Sample analysis was carried out under the best conditions.

## 3. Results and Discussion

### 3.1. Morphological and Structural Characterizations

**Figure 1:**
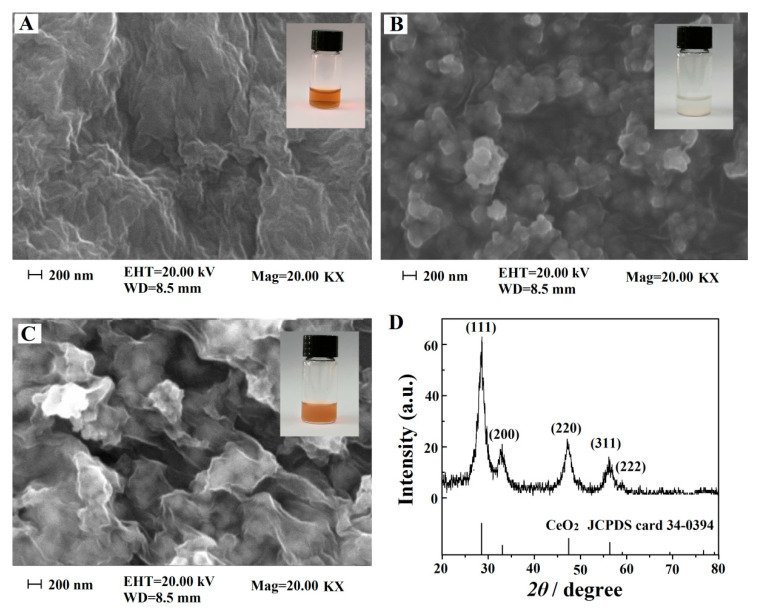
SEM images of (**A**) GO, (**B**) CeO_2_, (**C**) CeO_2_/ERGO nanocomposite and(**D**) the XRD pattern of CeO_2_. Inset: digital camera images of the pure GO, CeO_2_ and CeO_2_/ERGO nanocomposite.

The surface morphology of GO nanosheets, CeO_2_ nanoparticles, and CeO_2_/ERGO nanocomposites were studied by SEM. As shown in Figure 1A, the GO layer produced a rough, wrinkled and folded surface. The typical morphology of CeO_2_ nanoparticles is shown in Figure 1B. Most of CeO_2_ nanoparticles were spherical with a particle size distribution of about 20–200 nm. On the contrary, when CeO_2_ and ERGO were mixed together (Figure 1C), it was observed that CeO_2_ nanoparticles were wrapped inside by gossamer like ERGO, indicating that CeO_2_ and ERGO are well combined. The combination of CeO_2_ and ERGO provides a rich conductive channel for the electron transfer of Trp on the electrode surface. The XRD pattern of CeO_2_ is shown in Figure 1D. The diffraction peaks located at 2θ = 28.6°, 33.0°, 47.3°, 56.2° and 59.2° can be easily indexed to (111), (200), (220), (311), and (222) crystal planes of cubic fluorite structure of CeO_2_ [JCPDS 34-0394] [37].

**Figure 2:**
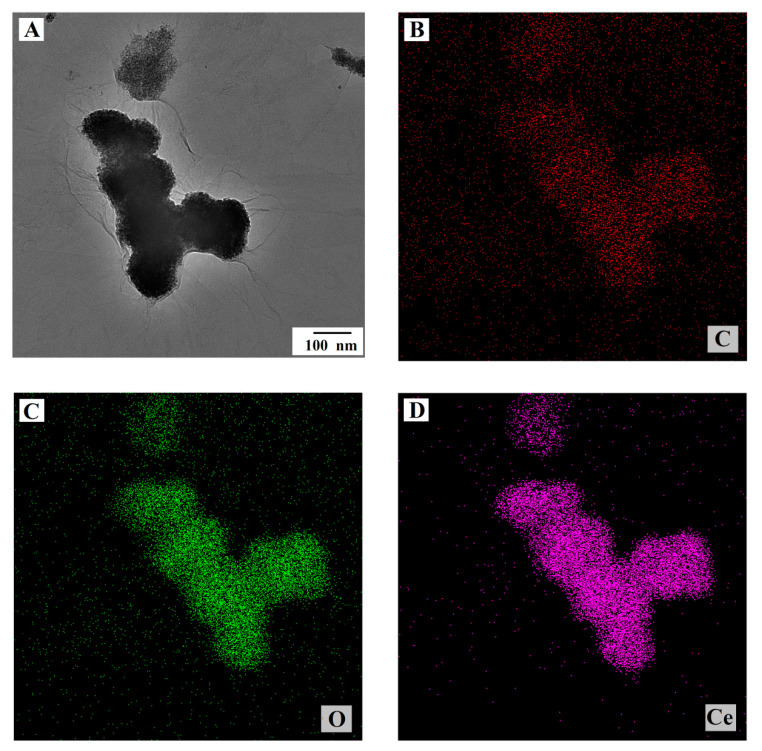
(**A**) TEM image of CeO_2_-ERGO nanocomposite; (**B**–**D**) Element mapping images of C, O and Ce of CeO_2_-ERGO composites.

The morphology of the as-prepared CeO_2_/ERGO nanocomposite was further examined by TEM. Figure 2A showed the TEM micrograph of the composite. It can be seen that most CeO_2_ nanoparticles were well wrapped in the ERGO sheets, and no free CeO_2_ nanoparticles were observed outside of the ERGO sheets, indicating a good interfacial interaction between the CeO_2_ nanoparticles and the ERGO sheets. The element composition and distribution of CeO_2_-ERGO nanocomposite were analyzed by EDS. As shown in Figure 2B–D, C, O, and Ce elements were observed in the element mapping images of the CeO_2_/ERGO composite. It was worth noting that the three elements were uniformly distributed throughout the CeO_2_/ERGO composite, indicating that the electrochemical reduction method is efficient to synthesize the CeO_2_/ERGO nanocomposite.

### 3.2. Electrochemical Characterization of the Modified Electrodes

**Figure 3:**
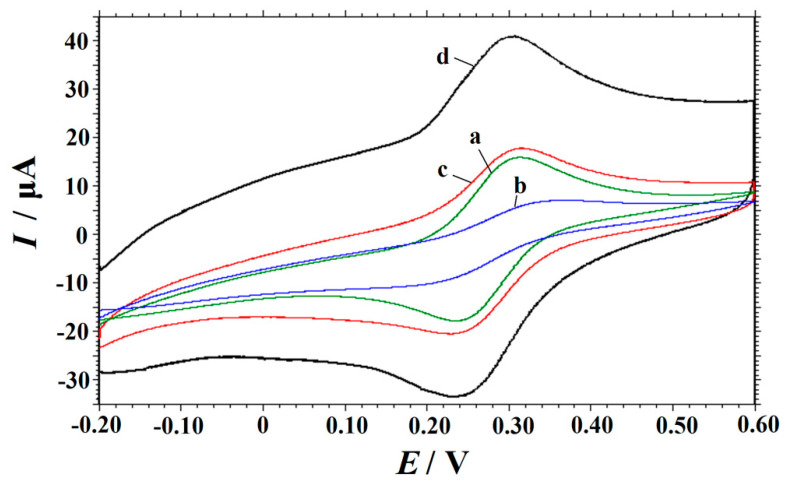
CVs of 1.0 mM K_3_[Fe(CN)_6_] in 0.5 M KCl recorded at (**a**) GCE, (**b**) GO/GCE, (**c**) CeO_2_/GCE and (**d**) CeO_2_/ERGO/GCE at a scan rate of 0.1 V s^−1^.

Using 1.0 mM K_3_[Fe(CN)_6_] as a probe, The properties of the surface of different electrodes were characterized by CV. Figure 3 exhibited the CV results obtained on bare GCE (**a**), GO/GCE (**b**), CeO_2_/GCE (**c**), and CeO_2_/ERGO/GCE (**d**) in the potential range of −0.2~0.6 V. On bare GCE a pair of well-defined redox peaks was observed with the peak-to-peak potential separation (Δ*E*_p_) of 85 mV and the redox peak current of 14.80 μA (*I*_pa_) and 15.87μA (*I*_pc_) (curve a). It was shown that the electron transfer process is quasi-reversible. However, the redox peak current decreased obviously on GO/GCE (*I*_pa_ =3.994 μA, *I*_pc_ =4.016 μA), Δ*E*_p_ was 167 mV (curve b). This may be due to the poor conductivity of GO and the strong negative charge repulsion force between [Fe(CN)_6_]^3−/4−^ and the ionized groups such as COO^−^ in GO. On the other hand, the current response of [Fe(CN)_6_]^3−/4−^ on the CeO_2_/GCE (curve c) increased slightly compared with that of bare GCE. On CeO_2_/ERGO/GCE (curve d), the Δ*E*_p_ value decreased to 78 mV and the highest redox peak current was observed, suggesting a more reversible electron transfer process of [Fe(CN)_6_]^3−/4−^ occurred on CeO_2_/ERGO/GCE. Therefore, the electron transfer rate was greatly improved due to the coexistence of ERGO and CeO_2_ nanoparticles.

### 3.3. Electrochemical Oxidation of Vanillin at Different Electrodes

**Figure 4:**
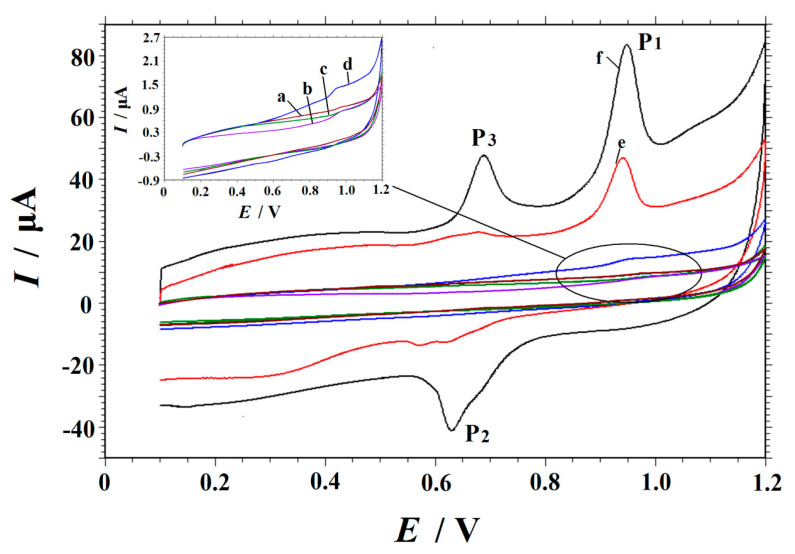
CVs obtained on different electrodes in 0.1 M HCl solution containing 10 μM vanillin (a: bare GCE; b: GO/GCE; c: CeO_2_/GCE; d: CeO_2_/GO/GCE; e: ERGO/GCE; f: CeO_2_/ERGO/GCE). Scan rate: 0.1 V·s^−1^.

**Scheme 2:**
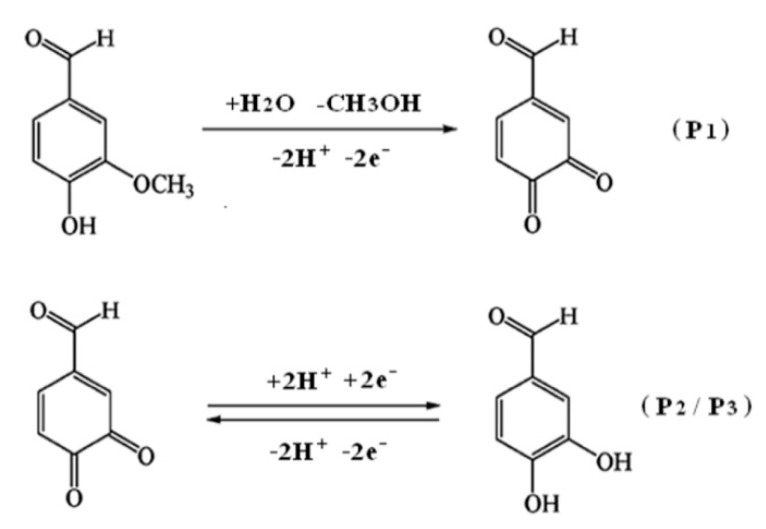
Oxidation mechanism of vanillin on the CeO_2_/ERGO/GCE.

The catalytic properties of CeO_2_/ERGO/GCE for vanillin oxidation are confirmed in Figure 4, which showed the CV responses of 10 μM vanillin recorded at different electrodes in 0.1 M HCl solution. On the CeO_2_/ERGO/GCE, a well-defined and sensitive vanillin oxidation peak (P_1_) appeared at 0.942 V [12,13]. In addition to the oxidation peak at 0.942 V, a redox couple P_2_/P_3_ (*E*_pa_ = 0.688 V, *E*_pc_ = 0.630 V) was also observed, corresponding to the redox reaction of the oxidized intermediate of vanillin. The electrochemical reaction mechanism was illustrated in Scheme 2 [12,13]. Table 1 compared the electrochemical data of vanillin obtained on different electrodes. It can be clearly observed that an obvious oxidation peak (*I*_p_ = 25.24 μA) appeared at 0.949 V at the ERGO/GCE compared with other electrodes, which may be related to the good catalytic activity, high conductivity, and large surface area of ERGO. The maximum oxidation peak current was obtained at CeO_2_/ERGO/GCE, which is about two times higher than that of ERGO/GCE, demonstrating the synergistic effect of CeO_2_ nanoparticles and ERGO nanosheets were existed on the electrode surface. As shown in Table 1, the order of the ability to enhance the oxidation signals of vanillin is CeO_2_/ERGO/GCE > ERGO/GCE > CeO_2_/GO/GCE > GO/GCE > CeO_2_/GCE > bare GCE. In particular, ERGO nanosheets have excellent conductivity and high surface area. Also the electron exchange between CeO_2_ nanoparticles and vanillin promoted the electrocatalytic reaction. In addition, ERGO is a good supporter for CeO_2_ nanoparticles, which can effectively prevent the agglomeration of CeO_2_ nanoparticles and give full play to its catalytic property. Therefore, the interface conductivity of the modified electrode was greatly improved and the sensitivity of the determination of vanillin was improved.

### 3.4. Effect of Scan Rate

**Figure 5:**
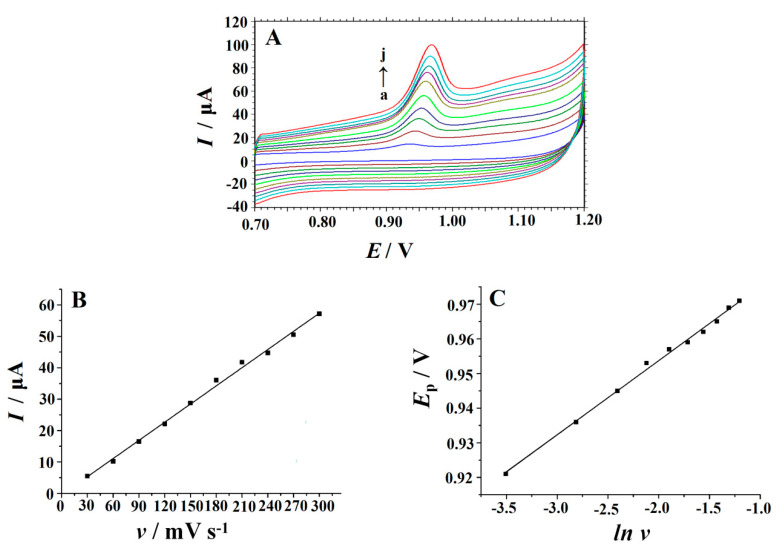
(**A**) CVs recorded at the CeO_2_/ERGO/GCE in 10 μM vanillin solution at different scan rates (a-j: 0.03, 0.06, 0.09, 0.12, 0.15, 0.18, 0.21, 0.24, 0.27, 0.30 V s^−1^); (**B**)The dependence of peak current on scan rate (*v*); (**C**) The dependence of peak potential on ln*v*.

In order to further investigate the oxidation mechanism, the CVs of 10 μM vanillin recorded on the CeO_2_/ERGO/GCE under different scan rates were illustrated in Figure 5A. Obviously, the oxidation peak increased gradually with the increase of scan rate in the range of 0.03–0.3 V s^−1^. A good linear relationship between the peak current (*I*_p_) and scan rate (*ν*) was observed in Figure 5B. The corresponding regression equation was *I*_p_ (μA) = 0.1929 *v* (V s^−1^) − 0.4991 (R = 0.9959), It shows that the oxidation process of vanillin on the CeO_2_/ERGO/GCE is controlled by adsorption. By plotting the relationship between logarithm *I*_p_ and logarithm *v*, the adsorption control behavior of vanillin was further confirmed: log *I*_p_ (μA) = 1.0371log*v* (V s^−^^1^) + 2.3049 (R = 0.9987). The obtained slope of 1.0371 is close to 1.0. In Figure 5C, it is worth noting that the *E*_p_ of vanillin moved to a positive value with the increase of scan rate, and *E*_p_ changed linearly versus Napierian logarithm of scan rate (ln *v*). The linear regression equation was *E*_p_ (V) = 0.0214 ln *v* (V s^−1^) + 0.9966 (R = 0.9981). For a completely irreversible adsorption control process, *E*_p_ can be expressed by the Laviron equation [43]:

*E_p_* = *E*^0^′ + (*RT/αnF*) ln (*RTk*^0^*/αnF*) + (*RT/αnF*) ln*v* (1)

According to equation (1), the slope of the straight line is equal to *RT/αnF*, so *αn* was calculated as 1.196. α is approximately 0.5 in a completely irreversible electrode process, thus *n* is about 2, which is consistent with the results obtained on silver nanoplate/GR composite modified GCE [12] and graphene-polyvinylpyrrolidone modified acetylene black paste electrode [13].

### 3.5. Chronocoulometric Studies

**Figure 6:**
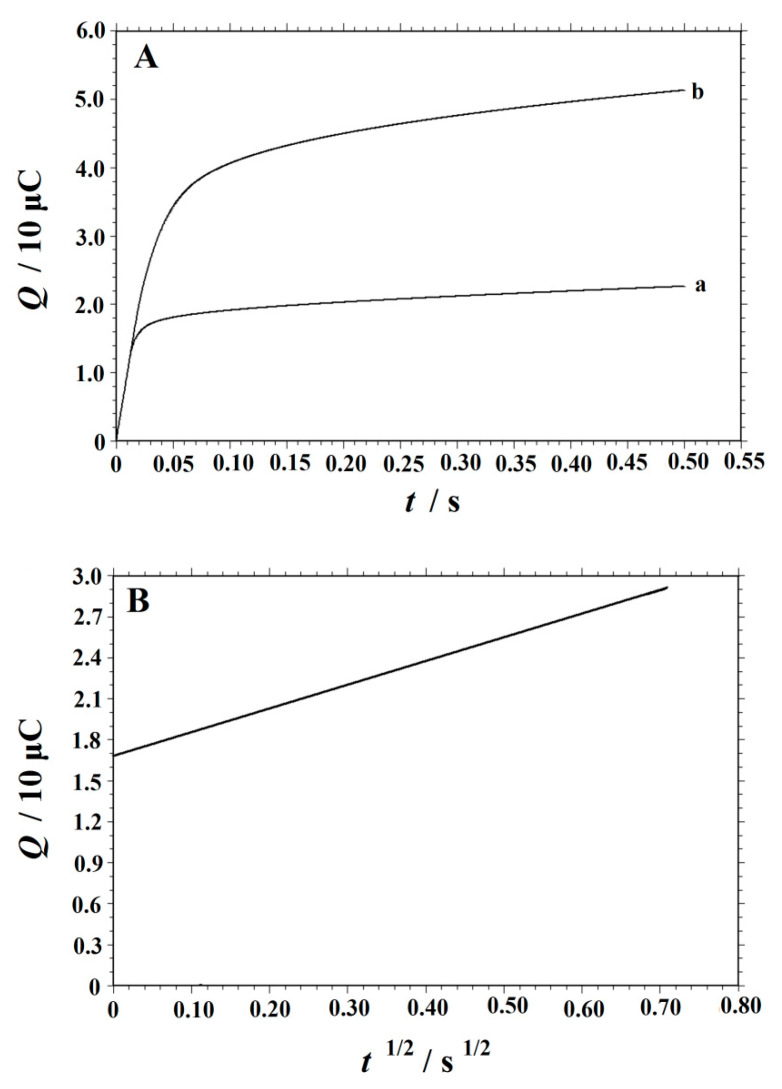
(**A**) Plot of *Q*–*t* curve obtained on the CeO_2_/ERGO/GCE in 0.1 M HCl solution in the absence (a) and presence (b) of 0.1 mM vanillin. (**B**) plot of *Q*–*t*^1/2^ curve on the CeO_2_/ERGO/GCE (background subtracted).

Using chronocoulometry, the diffusion coefficient *D* and Faradic charge *Q*_ads_ of vanillin at the CeO_2_/ERGO/GCE were calculated. Figure 6A showed the plot of *Q*–*t* obtained in the 0.1 M HCl solution with and without 0.1 mM vanillin. After background subtraction and the plot of charge (*Q*) against the square root of time (*t*^1/2^) (Figure 6B), a good linear relationship was observed with a slope of 1.729 × 10^−5^ C s^−1/2^ and *Q*_ads_ of 1.679 × 10^−5^ C. Using Equation (2) given by Anson [44], *D* can be obtained:

*Q* = 2*nFAcD*^*1*/2^ π^−1/2^*t*^1/2^+ *Q_dl_+ Q_ads_* (2)

where *Q_dl_* is double layer charge, *A* is the electrode surface area, and *c* is the substrate concentration. According to Figure 3, using Randles–Sevcik Equation (3) [45], *A* was estimated to be 0.01478 cm^2^.

*I*_p_ = 2.69 × 10^5^*n*^3/2^*A*_eff_*D*^1/2^*v*^1/2^*C* (3)

Thus, *D* of 2.9 × 10^−5^ cm^2^ s^−1^ was obtained. According to the equation *Q*_ads_ = *nFA Γ*_s_, the adsorption capacity *Γ*_s_ was obtained as 5.88 × 10^−10^ mol cm^−2^.

For the completely irreversible oxidation of vanillin at the CeO_2_/ERGO/GCE, the standard heterogeneous rate constant (*k*_s_) can be obtained based on Equation (4) [46,47]:

*k*_s_= 2.415 exp (−0.02*F/RT*) *D*^1/2^(*E*_p_− *E*_p/2_)^−1/2^*v*^1/2^ (4)

In the formula, *E*_p_ represents the peak potential, *E*_p/2_ is the potential at which *I* = *I*_p_/2. Other symbols have their usual meanings. In our experiment, *E*_p_ − *E*_p/2_ =34 mV, *D* = 2.9 × 10^−5^ cm^2^ s^−1^, *v* = 100 mV s^−1^, and T = 298 K. The value of *k*_s_ was obtained as 1.02 × 10^−2^ cm s^−1^, confirming a relative rapid electrode reaction process.

### 3.6. Optimal of Some Determination Conditions

**Figure 7:**
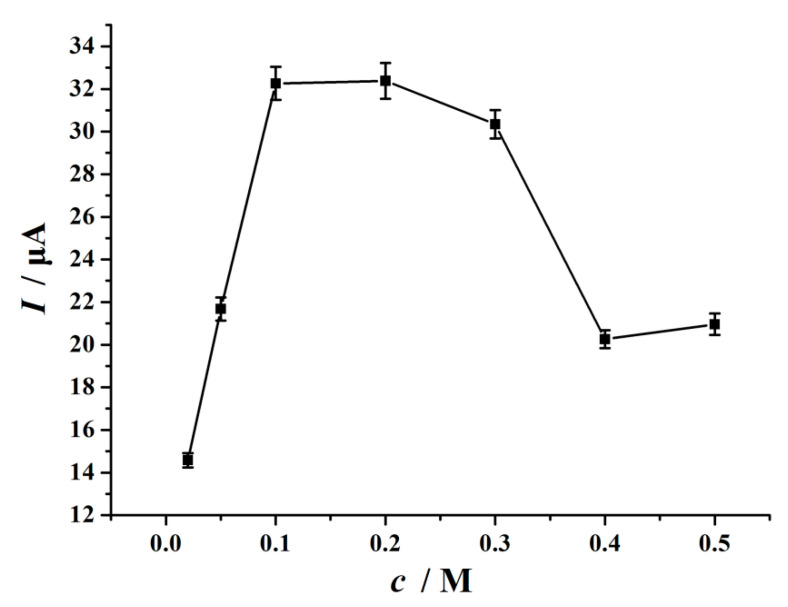
Effect of HCl concentration on the peak current of 10 µM vanillin at CeO_2_/ERGO/GCE.

The electrochemical response of vanillin on the CeO_2_/ERGO/GCE was investigated in different types of supporting electrolytes, such as HAc–NH_4_Ac buffer solution (pH 4.0), phosphate buffer solution (pH 3.0 and 6.0), HAc–NaAc buffer solution (pH 4.0), HCl, H_2_SO_4_, HNO_3_, and H_3_PO_4_ (each 0.1 M). It was found that the peak current was the largest and the peak shape was sharp in HCl solution. In the range of 0.02–0.5 M, the effect of HCl concentration on the peak current of 10 μM vanillin was studied. It was found that the maximum peak current of vanillin was obtained when the concentration of HCl reached 0.1–0.2 M (Figure 7). In this experiment, 0.1 M HCl solution was selected for vanillin detection.

**Figure 8:**
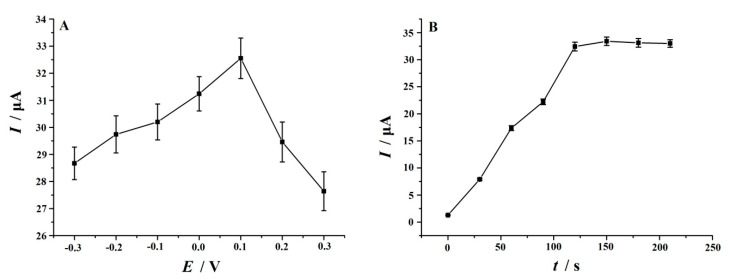
(**A**) The effect of accumulation potential (**A**) and accumulation time (**B**) on the peak current. Vanillin concentration: 10 µM, supporting electrolyte: 0.1 M HCl, scan rate: 0.1 V s^−1^.

Since the electrode process of vanillin on the CeO_2_/ERGO/GCE is controlled by adsorption, the accumulation conditions have a great influence on the peak current of vanillin. As showed in Figure 8A, when the accumulation potential changed in the range of −0.3–0.3 V, the peak current of vanillin increased first and then decreased, and reached the maximum value at 0.1 V. As shown in in Figure 8B, the peak current was greatly affected by the accumulation time. In the initial 120 s, the peak current increased significantly, and then the peak current tended to be stable when the time exceeded 120 s. This may be due to the saturated adsorption of vanillin. Therefore, the accumulation conditions (0.1 V, 120 s) were selected for vanillin detection.

### 3.7. Linear Range and Detection Limit

**Figure 9:**
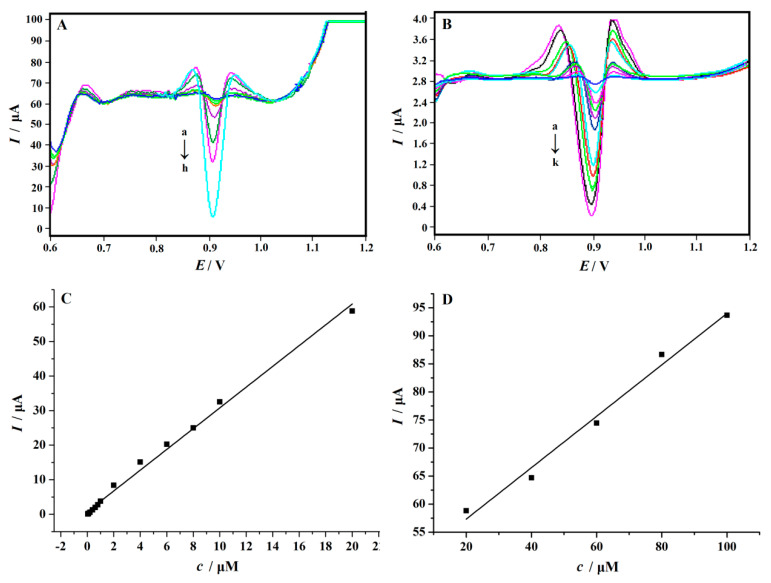
SDLSVs of different concentration of vanillin in 0.1 M HCl solution recorded at the CeO_2_/ERGO/GCE after accumulation at 0.1 V for 120 s at a scan rate of 0.1 V s^−1^. (**A**) a-h: 0.04, 0.06, 0.08, 0.1, 0.2, 0.4, 0.6, 0.8 μM; (**B**) a-k: 1, 2, 4, 6, 8, 10, 20, 40, 60, 80, 100 μM. (**C**) Calibration curve (*C*_vanillin_ = 0.04–20 μM) and (**D**) Calibration curve (*C*_vanillin_ = 20–100 μM).

Compared with the traditional CV method, the second-order derivative linear sweep voltammetry (SDLSV) has the advantages of low detection limit and high sensitivity, and is a more effective electrochemical technique for quantitative analysis. Therefore, SDLSV was used for the determination of vanillin in this study. Figure 9A,B showed the voltammetric curves of vanillin at different concentrations on the CeO_2_/ERGO/GCE under the optimized conditions. The proportional of well-defined peak current to vanillin concentration was observed in Figure 9C,D and the calibration curve in the form of *I*_p_ versus concentration of vanillin showed two linear regions from 0.04 to 20 μM and 20–100 μM with the equations of *I* (μA) = 3.003*c* (μM) + 0.7587 (R = 0.9941) and *I* (μA) = 0.4582*c* (μM) + 48.159 (R = 0.9836), respectively. The detection limit was 0.01 μM. In order to estimate the analytical characteristics of the developed sensor, the CeO_2_/ERGO/GCE was comprehensively compared with other published electrochemical methods for vanillin detection. From the results in Table 2, it was found that, compared with other electrodes, the developed CeO_2_/ERGO/GCE has better detection performance with a wider dynamic range and lower detection limit.

### 3.8. Reproducibility, Stability and Selectivity of CeO_2_/ERGO/GCE

**Figure 10:**
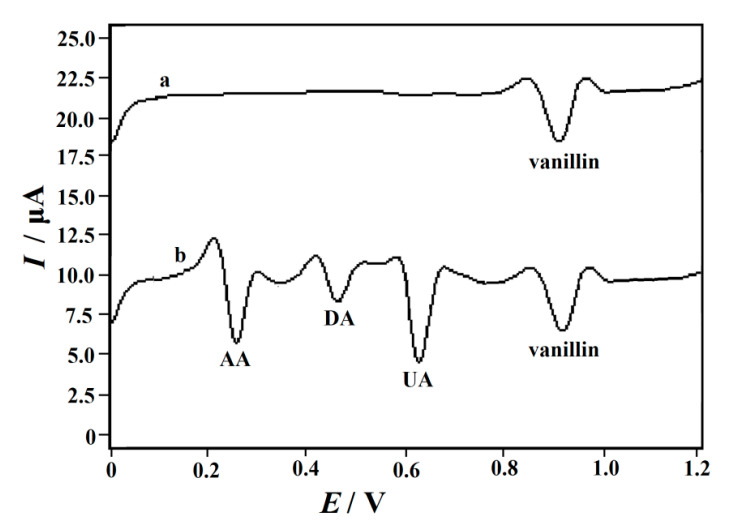
SDLSVs obtained at the CeO_2_/ERGO/GCE in 0.1 M HCl solution containing (a) 1.0 μM vanillin; (b) 1.0 mM AA+ 10 μM DA + 10 μM UA + 1.0 μM vanillin. Accumulation potential: 0.1 V, accumulation time: 120 s, scan rate: 0.1 V s^−1^.

Seven CeO_2_/ERGO/GCEs were fabricated by the same method to test the reproducibility, and 10 μM vanillin solution was measured under the same conditions. The relative standard deviation (RSD) of 4.62% (n = 7) indicated excellent reproducibility. In addition, to test the repeatability, the vanillin solution was determined by a single CeO_2_/ERGO/GCE for 10 times. After each measurement, to regenerate the electrode surface, two successive voltammetric sweeps were carried out in 0.1 M H_2_SO_4_ solution in the range of 0.2–1.2 V. The good repeatability was reflected by the RSD of 2.74%. The storage stability of the modified electrode was also evaluated by storing the modified electrode in air. After two weeks, the response of the electrode is still 91.6% of the initial current, which showed that the electrode has long-term stability. In addition, it was found that when a 1000-fold amount of sucrose, glucose, fructose, K^+^, Ca^2+^, Na^+^, Mg^2+^, Zn^2+^, Al^3+^, and a 500-fold amount of citric acid, tartaric acid, lactic acid, and caffeine were present, the peak current change of 10 μM vanillin was less than 5%. Ascorbic acid (AA), uric acid (UA) and dopamine (DA) as three kinds of important biological substances in the human fluid. Figure 10 exhibited the SDLSVs obtained at the CeO_2_/ERGO/GCE in the presence of 1.0 mM AA, 10 μM DA, 10 μM UA, and 1.0 μM vanillin. As shown in Figure 10, four oxidation peaks were well separated in 0.1 M HCl solution, the oxidation peak potentials of AA, DA, UA and vanillin were 0.248 V, 0.473 V, 0.620 V, and 0.918 V, respectively. It was found that there was no obvious interference for the oxidation signal of vanillin (signal change below ±5%).

### 3.9. Application

The determination of vanillin in commercial food products such as biscuit, chocolate and pudding powder was performed using the developed method. The sample solutions were prepared according to reference [13], and the results were listed in Table 3. The contents of vanillin in these samples were calculated as 46.25 μg g^−^^1^ (for biscuit), 69.38 μg g^−^^1^ (for chocolate) and 132.98 μg g^−^^1^ (for pudding powder), respectively. The recoveries were 97.0–104.0%, which showed that the method can be used for the accurate and rapid detection of vanillin in commercial food samples.

## 4. Conclusions

In this paper, CeO_2_ nanoparticles were synthesized by the hydrothermal method and dispersed uniformly in graphene oxide (GO) aqueous solution by ultrasonication. The homogeneous CeO_2_-GO dispersion was coated on the surface of a glassy carbon electrode (GCE), and GO was converted into ERGO by potentiostatic reduction to obtain the modified electrode (CeO_2_/ERGO/GCE). This method offers several advantages over other techniques, including being green, efficient, inexpensive, and rapid. The electrochemical behaviors of vanillin on the CeO_2_/ERGO/GCE were studied carefully and the electrochemical parameters were calculated. The obtained CeO_2_/ERGO nanocomposite exhibited excellent performance for vanillin oxidation due to its strong electrocatalytic ability, good conductivity, and large surface area. Using the SDLSV technique, vanillin can be detected in the concentration range of 0.04–20 μM and 20–100 μM with the detection limit of 0.01 μM. Furthermore, the developed method has the advantages of high sensitivity, good selectivity, simple electrode preparation, and low cost. It has a wide application prospect in the sensitive detection of vanillin in commercial foods.

## Figures and Tables

**Table 1:** Detailed data of CVs of 10 µM vanillin recorded at different electrodes.

Electrode	*I*_p_/μA	*E*_p_/V
GCE	0.3242	0.978
GO/GCE	0.6226	0.979
CeO_2_/GCE	0.3469	0.980
CeO_2_/GO/GCE	1.146	0.953
ERGO/GCE	25.24	0.949
CeO_2_/ERGO/GCE	52.22	0.942

**Table 2:** Comparison of properties of different modified electrodes for the detection of vanillin.

Electrochemical Sensors	Technique	Linear Range /μM	Detection Limit /μM	References
^a^ AuPd-GR/GCE	^k^ DPV	0.1–7; 10–40	0.02	[9]
^b^ CoS NR@Nafion/GCE	DPV	0.5–56	0.07	[10]
^c^ MoS_2_-CNF/GCE	amperometry	0.3–135	0.15	[11]
^d^ Ag NPs/GN/GCE	^l^ SWV	2–100	0.332	[12]
^e^ GR-PVP/ABPE	SDLSV	0.02–2.0; 2.0–40; 40–100	0.01	[13]
^f^ BDD electrode	^m^ SWASV	3.3–330	0.38	[14]
^g^ APT-BDD	SWASV	6.6–660	1.47	[15]
^h^ CTABMGPE	DPV	4–15; 20–70	1.29	[16]
^i^ Ag-Pd/GO/GCE	DPV	0.02–45	0.005	[17]
^j^ MWNTs-PDA@ MIP/SWNTsCOOH/GCE	DPV	0.2–10	0.1	[18]
CeO_2_-ERGO/GCE	SDLSV	0.04–20; 20–100	0.01	This work

^a^ Au-Pd nanoparticles−graphene/GCE; ^b^ CoS nanorods (NR) @Nafion/GCE; ^c^ electrospun molybdenum disulfide/carbon nanofibers/GCE; ^d^ silver nanoplates/grapheme/GCE; ^e^ graphene–polyvinylpyrrolidone/GCE; ^f^ cathodically pretreated boron-doped diamond electrode; ^g^ anodically pretreated boron-doped diamond electrode; ^h^ ionic surfactant modified graphene paste electrode; ^i^ Ag-Pd bimetallic nanoparticles/graphene oxide/GCE; ^j^ molecularly imprinted poly(1-vinyl-3-octylimidazole hexafluoride phosphorus)−multi-walled carbon nanotubes@polydopamine–carboxyl single-walled carbon nanotubes composite/GCE; ^k^ differential pulse voltammetry; ^l^ square-wave voltammetry; ^m^ square-wave adsorptive stripping voltammetry.

**Table 3:** Determination results of vanillin in food samples.

Samples	Added/μM	Found/μM	RSD/%	Recovery/%
Biscuit	0	1.52	2.86	-
	1.0	2.56	3.19	104.0
	2.0	3.48	2.14	98.0
Chocolate	0	2.28	2.37	-
	1.0	3.25	2.75	97.0
	2.0	4.32	2.84	102.0
Pudding powder	0	4.37	2.16	-
	2.0	6.44	2.51	103.5
	4.0	8.30	2.83	98.3
